# A novel system for predicting liver histopathology in patients with chronic hepatitis B

**DOI:** 10.1097/MD.0000000000006465

**Published:** 2017-04-07

**Authors:** An-Na Pan, Wang-Wang Xu, Yun-Lin Luo, Huan-Huan Yu, Yi-Bing Hu, Qing-Feng Sun, Ji-Guang Ding, Yang-He Wu

**Affiliations:** a Department of Infection, The Third Affiliated Hospital of Wenzhou Medical University, Ruian; b Department of Infection, Wenzhou People's Hospital; c Wenzhou Medical University, Wenzhou, Zhejiang Province, China.

**Keywords:** chronic hepatitis B, liver biopsy, liver fibrosis, liver histology, liver inflammation

## Abstract

There is currently a lack of reliable, reproducible, and easily applied methods for assessing changes in liver histology in patients in the gray zone phase of chronic hepatitis B (CHB). Therefore, we aimed to develop a novel predictive scoring system to detect significant liver histological changes in these patients.

A total of 388 patients in the gray zone phase of CHB who underwent liver biopsy were divided into a training group and a validation group, and their clinical and routinely available laboratory parameters were analyzed using univariate analysis, Spearman correlation analysis, and logistic modeling. A novel scoring system, termed the Significant Histological Model (SHM), was constructed using logistic modeling. The diagnostic accuracy of our novel scoring system was evaluated by the receiving operating characteristic (ROC) method, sensitivity, specificity, and positive and negative predictive values (NPVs).

We established the novel SHM scoring system using serum aspartate transaminase (AST), platelet counts (PLTs), albumin (ALB), and hepatitis B virus (HBV) DNA (log_10_ IU/mL) levels. The area under the ROC curve of the SHM scoring system was 0.763 in the training group and 0.791 in the validation group. For patients with a score of −1.0 or less and no significant histological changes, the sensitivity was 78.9%, specificity was 51.5%, positive predictive value (PPV) was 46.4%, and NPV was 82.0%. In the validation set, the sensitivity, specificity, PPV, and NPV were 80.0%, 66.6%, 56.3%, and 86.2%, respectively.

This novel scoring system using AST, PLT, ALB, and HBV DNA (log_10_ IU/mL) levels identifies patients in the gray zone phase of CHB with and without histological changes with a high degree of accuracy. Here, we provide the experimental basis for the initiation of clinical antiviral treatment without the need for liver biopsy.

## 1 Introduction

Chronic hepatitis B (CHB) virus infection, which infects an estimated 240 million people worldwide,^[1]^ is a major public health issue that mostly affects resource-limited countries. The World Health Organization estimates that 1 million patients with hepatitis B virus (HBV) worldwide die each year of complications of liver cirrhosis, namely liver failure and hepatocellular carcinoma (HCC).^[2]^ HBV covalently closed circular DNA serves as a viral replication template and plays a role in HBV infection persistence in the DNA of liver cells. Thus, antiviral treatment can reduce the risk of CHB progression to cirrhosis or HCC.^[3]^

The guidelines of the American, European, and Asian societies for liver research (AASLD, EASL, and APASL, respectively)^[4–6]^ recommend starting with antiviral treatment based on alanine transaminase (ALT) and HBV DNA levels. Patients with an ALT lower than 2 times the upper limit of normal (ULN) are not advised to start treatment. Several groups^[7–9]^ have reported that patients with ALT levels 1- to 2-fold higher than the ULN, particularly those >40 years of age, may have liver inflammation and fibrosis that can lead to cirrhosis, HCC, or other end-stage liver disease. We believe that these patients are in the “gray zone” phase of the disease as recently reported by Yapali et al.^[10]^

Patients in the gray zone of HBV often undergo liver biopsy for histopathological diagnosis and evaluation of inflammation and fibrosis staging. Based on international guidelines,^[4–6]^ antiviral treatment is required for patients with moderate to severe inflammation or fibrosis. Thus, liver biopsy currently remains the only reliable method for liver fibrosis and inflammation staging. However, it is invasive and carries several risks of complications.^[11–13]^ Moreover, patients are not willing to undergo repeated biopsies to monitor disease progression. Transient elastography (FibroScan) has shown excellent accuracy in the diagnosis of fibrosis^[14–18]^ and gained approval for its clinical use in the US. However, it does not provide information about intrahepatic inflammation, which is of utmost importance when choosing a specific antiviral therapy. Furthermore, it is expensive and limited for several reasons. Many noninvasive methods have been created for predicting liver fibrosis and inflammation. However, these procedures are not widely available and are expensive. Additionally, methods to evaluate patients in the gray zone of CHB have not yet been established.

The aim of this study was to construct a reliable model using routine serum markers directly related with liver histological changes in patients with gray-zone CHB. Here, we describe our novel scoring system for predicting liver pathological changes in these patients.

## 2 Methods

### 2.1 Patients

We retrospectively enrolled 388 patients with CHB who underwent percutaneous liver biopsy and blood tests at Beijing Ditan Hospital between June 2009 and January 2013. The shortest time of the patients carrying hepatitis B surface antigen was 6 months, and the lowest HBV DNA level in these patients was 500 IU/mL. We chose patients with the following characteristics: >40 years of age, normal, or abnormal serum ALT levels less than 2-fold higher than the ULN (our laboratory reference value is 40 IU/L). Patients who met 1 or more of the following conditions were excluded: alcoholic liver disease, nonalcoholic fatty liver disease, autoimmune hepatitis, compensated or decompensated cirrhosis, renal insufficiency, other causes of chronic liver disease, coinfection with human immunodeficiency virus or hepatitis C virus, insufficient amount of donor living tissue, or incomplete clinical data. Before the liver biopsy, none of the patients received antiviral therapy. Written consent was obtained prior to liver biopsy, and all trials were approved by the ethics committee of Beijing Ditan Hospital.

### 2.2 Liver biopsy

The physicians who performed liver biopsies are employees of Beijing Ditan Hospital. Every patient underwent ultrasound-guided liver biopsy by a 16 G × 15 cm needle. The biopsy specimens were immediately fixed with formalin, embedded in paraffin, and processed with hematoxylin and eosin, Masson trichrome, and reticular fiber staining. For diagnostic purposes, the tissue was >1 cm long and covered at least 6 portal tracts. Fibrosis staging and inflammation grades were determined using the METAVIR scoring system: S0, S1, S2, S3, S4; G0, G1, G2, G3, and G4.

### 2.3 Laboratory tests

The serum samples and biochemical and virological data were collected within 1 week prior to liver biopsy. Blood tests were performed using an automatic biochemistry analyzer. Serum parameters included total bilirubin, ALT, aspartate aminotransferase (AST), γ-glutamyl transpeptidase, albumin (ALB), alkaline phosphatase, globulin, blood platelet count (PLT), prothrombin time activity (PTA), α-fetoprotein, and white blood cells. Serum HBV DNA levels and hepatitis Be antigen (HBeAg) were measured by a fluorescence quantitative polymerase chain reaction assay (PG Company, Shenzhen, China).

### 2.4 Statistical methods

Data were randomly divided into a training cohort and a validation cohort. The training cohort of 259 patients (66.8%) was used to develop the scoring system. The remaining 129 patients (33.2%) formed the validation cohort. SPSS 19.0 software was used to analyze the statistical data. Quantitative variables are expressed as mean values and categorical variables are expressed as number (percentage). Categorical data were compared using the Mann–Whitney *U* test. Mean values between the 2 groups were compared using Student *t* test. Values of *P* < 0.05 were considered statistically significant.

To formulate our predictive model, univariate analysis was performed of variables of patients with or without significant fibrosis in the training group. Significant variables from the univariate analysis (*P* < 0.05) were then subjected to Spearman correlation analysis and multivariate analysis by forward logistic regression to identify independent factors associated with significant histological changes. A predictive scoring system was constructed by modeling the values of the independent variables and their coefficients of regression. The diagnostic value of the scoring system was assessed using receiver-operating characteristic (ROC) curve analysis. Diagnostic accuracy was assessed by sensitivity, specificity, positive predictive value (PPV), and negative predictive value (NPV). The best cut-off points were selected from the ROC curve to identify the presence and absence of significant histological change. The new scoring system, derived from the training group, was then applied to the validation group to test for accuracy by measuring the areas under the ROC curves (AUROCs).

## 3 Results

A total of 388 patients and 15 parameters were included in the study, including 236 men and 152 women. Data were randomly divided into a training cohort and a validation cohort. A training cohort consisting of 259 patients (66.8%) was used to develop the model. The remaining 129 patients (33.2%) formed the validation cohort. Patient characteristics at the time of liver biopsy are shown in Table 1. There were no significant differences between the training and validation groups in any of the variables.

**Table 1:**
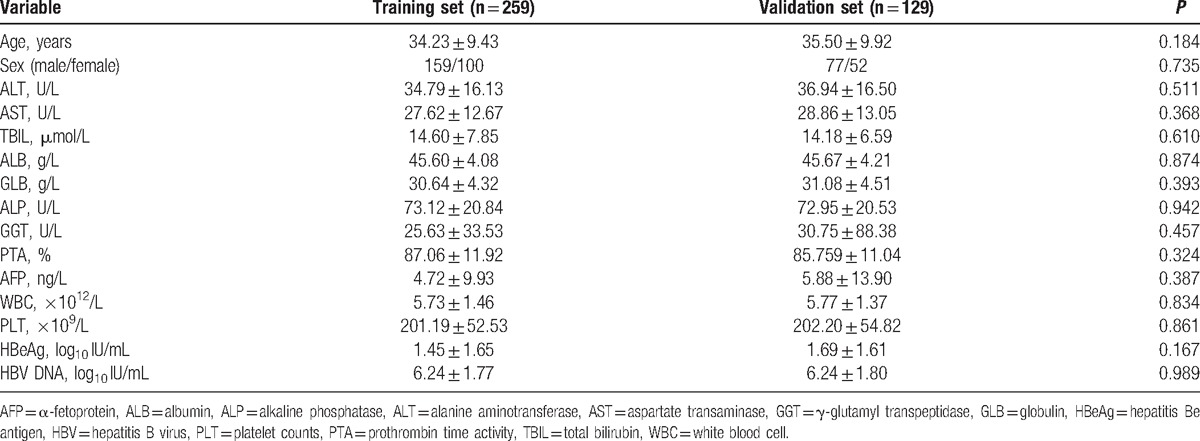
Baseline characteristics of patients in the training and validation sets.

Patients with high fibrosis (S > 2) and/or high inflammation (G > 2) were placed in the significant histological group, while those with low fibrosis (S < 2) and low inflammation (G < 2) were placed in the nonsignificant histological group (Table 2). After univariate analysis, ALT, AST, ALB, PLT, PTA, HBeAg (log_10_ IU/mL), and HBV DNA (log_10_ IU/mL) differed significantly between the mild and severe groups (*P* < 0.05). These markers were assessed by Spearman correlation analysis, and the following 7 variables were significantly correlated with the histological findings: ALT (*r* = 0.167; *P* < 0.05), AST (*r* = 0.306; *P* < 0.001), PLT (*r* = −0.241; *P* < 0.001), ALB (*r* = −0.170; *P* < 0.05), PTA (*r* = −0.126; *P* < 0.05), HBeAg (log_10_ IU/mL) (*r* = −0.111; *P* < 0.05), and HBV DNA (log_10_ IU/mL) (*r* = −0.199; *P* < 0.05). The variables associated with the presence of significant histological changes were assessed by unadjusted single-predictor logistic model analysis. Subsequent forward logistic regression analysis indicated that PLT (*P* = 0.029), AST (*P* = 0.000), ALB (*P* = 0.034), and HBV DNA (log_10_ IU/mL) (*P* = 0.001) were independent predictors of significant histological changes (Table 3).

**Table 2:**
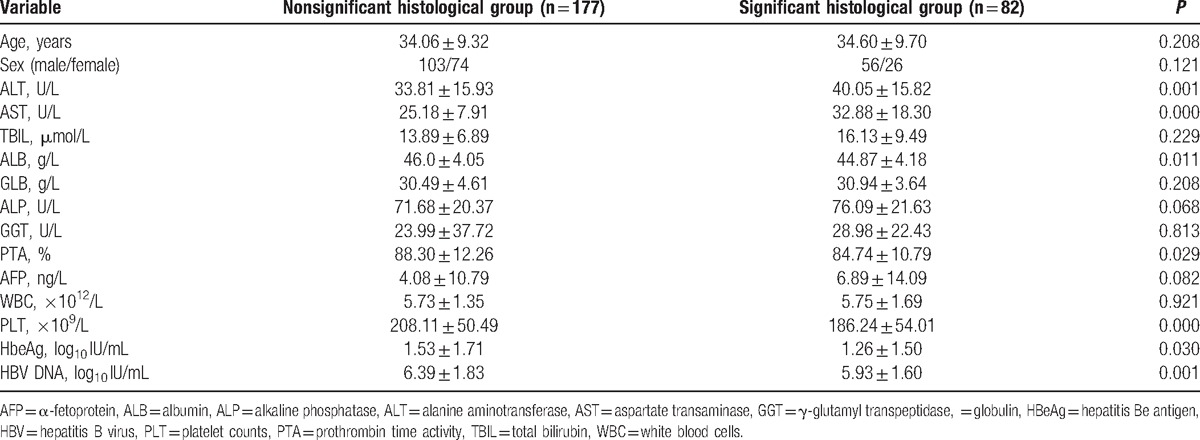
Characteristics of patients in the mild (n = 177) and severe (n = 82) groups.

**Table 3:**
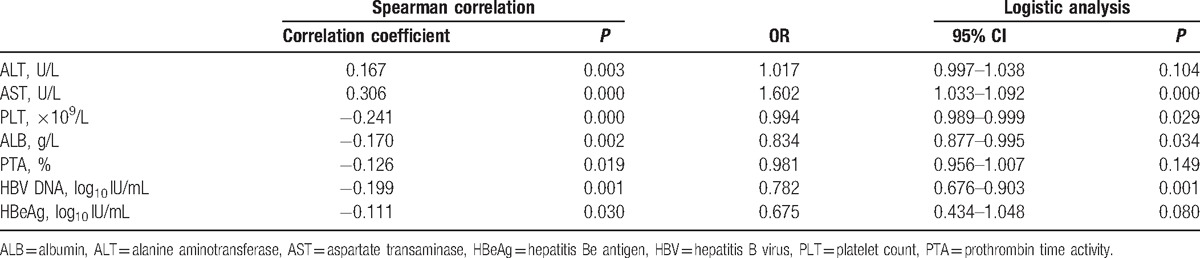
Spearman correlation analysis and logistic model analysis to identify independent predictors of significant histological changes.

**Figure 1:**
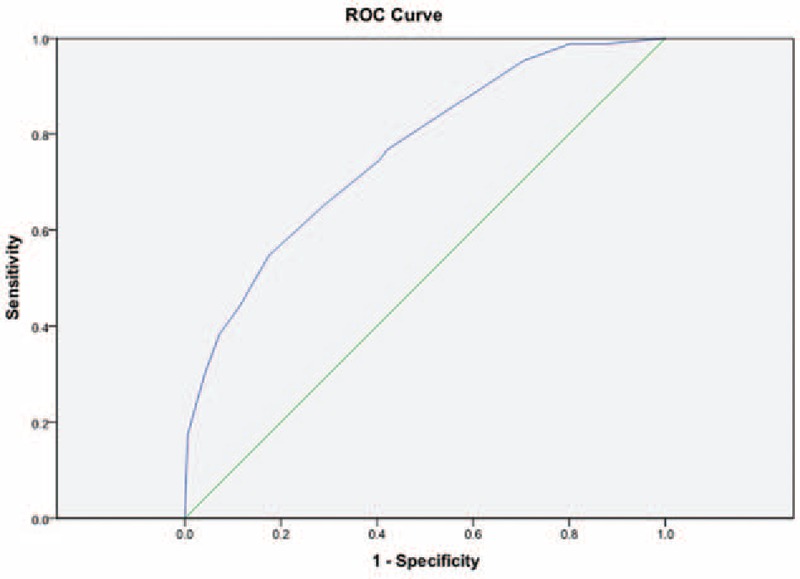
Diagnostic value of the new scoring system using receiver-operating characteristic curve analysis of the training set.

By modeling the values of the independent variables and their coefficient of regression, we constructed a new scoring system termed the Significant Histological Model (SHM) (logistic y = 3.339 + 0.06 × AST − 0.06 × PLT − 0.068 × ALB − 0.246 × HBV DNA [log10 IU/mL]). The calculated AUROC for predicting significant histological changes was 0.763 (Fig. 1). The optimal cut-off point was set as −1.0. For patients with a score of −1.0 or less and no significant histological changes, the sensitivity was 78.9%, specificity was 51.5%, PPV was 46.4%, and NPV was 82.0% (Table 4).

**Table 4:**
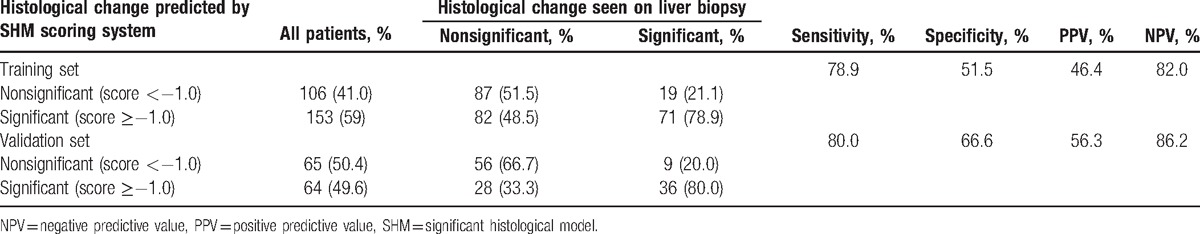
Predictive value of the SHM scoring system for the training and validation groups.

**Figure 2:**
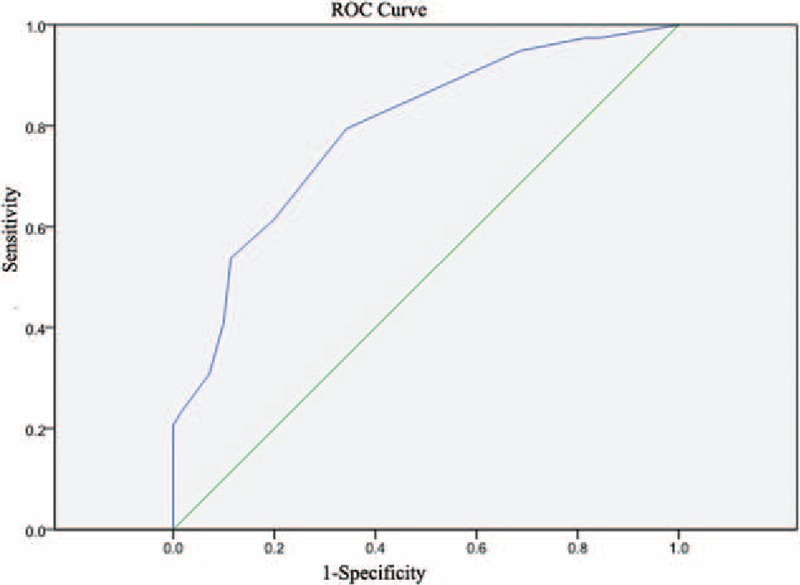
Diagnostic value of the new scoring system using receiver-operating characteristic curve analysis of the training set.

In the validation group, the calculated AUROC for predicting significant histological changes was 0.791 (Fig. 2). For patients with a score of −1.0 or less without significant histological changes, the sensitivity was 80.0%, specificity was 66.6%, PPV was 56.3%, and NPV was 86.2% (Table 4).

## 4 Discussion

Here, we aimed to develop a simple scoring system to predict the risk of significant histological changes (inflammation and fibrosis) for patients in the gray zone phase of CHB. This novel SHM scoring system consists of 4 independent predictors of significant liver pathological changes during the subsequent forward logistic regression analysis. We found that PLT, AST, ALB, and HBV DNA (log10 IU/mL) were independent predictors for significant histological changes in patients in the gray zone of CHB. Decreased PLT during the progression of liver fibrosis has been previously reported, and the PLT value correlated with advanced fibrosis.^[19,20]^ Thrombocytopenia is a demonstrated surrogate marker for liver cirrhosis,^[21,22]^ especially in hepatitis patients with viral infection. Moreover, AST and ALB usually correlate with cellular death within the liver. AST is located mainly in the mitochondria of liver cells. Moreover, ALB is produced in the liver. Liver dysfunction leads to increased AST and decreased ALB. Here, we found that patients with a high HBV DNA level were at low risk of significant liver pathological changes. This might be due to the fact that patients with a high HBV DNA level are likely in the immune tolerance period. These factors reflect that liver dysfunction and might be associated with liver pathological changes.

Using our SHM scoring system, we calculated an AUROC of 0.763 for predicting significant histological changes (inflammation and fibrosis) for patients in the gray zone phase of CHB in the training group and an AUROC of 0.791 in the validation group. The NPV of the optimal cut-off value of −1.0 in the training group and validation group was 82.0% and 86.2%, respectively. Using this value, 82.0% of the nonsignificant histological change patients in the training set were correctly identified, as were 86.2% of the nonsignificant histological change patients in the validation set. Therefore, we highly recommend that patients with a prediction score less than −1.0 not undergo antiviral treatment immediately or undergo liver biopsy, thus avoiding its associated risks and high cost. Moreover, clinicians should evaluate these patients regularly using our novel scoring system. The sensitivity of a cut-off value of −1.0 in the training and validation groups were 78.9% and 80.0%, respectively. Patients with scores >−1.0 are at a relatively high risk of significant pathological changes, which may lead to liver cirrhosis, HCC, or other end-stage liver disease. In these cases, we highly recommend starting immediately with antiviral treatment.

Although there is currently a lack of noninvasive diagnostic methods of liver fibrosis, markers for predicting liver inflammation are relatively scarce. The major strength of the current scoring system is that it can predict liver fibrosis and liver inflammation using a cut-off value of −1.0. In contrast, other noninvasive methods such as the AST to platelet ratio index,^[23]^ Lok index,^[24]^ Forn index,^[25]^ the FIB-4,^[26]^ and the Zeng score^[27]^ can only predict severe fibrosis. Another advantage of our scoring system is that it requires only a simple calculation. Our novel method requires only 4 indicators, so it can be applied by clinicians simply and easily. It is also a specific method established for patients in the gray zone of CHB to predict liver histological changes.

However, our study has limitations. First, the target population was gray-zone patients with CHB; thus, the tested application of our scoring system is rather limited. Second, the scoring system has not yet been compared with other noninvasive methods. Thus, it may require further validation prior to application in other clinical settings. Another limitation is that our normal ALT level was 0 to 40 U/L; however, recent studies have shown normal ALT levels of 30 U/L for men and 19 U/L for women ^[28]^.

Here, we reported a new scoring system based on 4 indicators: AST, PLT, ALB, and HBV DNA (log10 IU/mL), all of which are associated with significant histological changes in patients with gray-zone CHB. This novel scoring system identified patients in the gray zone phase of CHB without histological changes with a high degree of accuracy. This useful tool may provide an experimental basis for the initiation of clinical antiviral treatment as well as decrease the need for liver biopsy.
